# Giant tubular adenoma of the accessory breast in the anterior chest wall occurred in a pregnant woman

**DOI:** 10.1186/s13000-015-0286-0

**Published:** 2015-06-04

**Authors:** Yaoyu Huang, Hao Zhang, Qian Zhou, Lijun Ling, Shui Wang

**Affiliations:** Department of Breast Surgery, The First Affiliated Hospital with Nanjing Medical University, Nanjing, Jiangsu 210029 China; Department of Breast Surgery, Nanjing Maternity and Child Health Care Hospital Affiliated to Nanjing Medical University, Nanjing, Jiangsu China

**Keywords:** Tubular adenoma, Accessory breast, Pregnant woman

## Abstract

**Background:**

Tubular adenoma of the breast is a rare benign epithelial tumor and only a few literatures have been reported; so far, no cases of tubular adenoma occurred in the accessory breast have been reported in the English literature. Clinical presentation and management of our patient are discussed along with a review of the literature on accessory mammary and tubular adenoma.

**Case presentation:**

We present a case of 26-year-old woman (gravid 4, para 1) at 37 weeks of pregnancy with rapid enlargement in left anterior chest wall during pregnancy. Physical examination showed the left accessory breast was obviously bigger than the right one that only had a light areola around a small nipple. An elastic, mobile well-circumscribed mass measuring approximately 15 cm × 15 cm was palpated. Moreover, it was edematous and congestive with an increase in local temperature. The breast ultrasound further demonstrated the mass was a relatively homogeneous solid with short stripe blood flow signal. A single live fetus of 37 weeks gestation was observed by abdominal ultrasound scan. After a 2850 g male neonate was delivered, the right accessory breast and the mass in left accessory breast were removed. The resected specimen appeared as a solid white elastic mass with a smooth surface and the cut surface was red-grayish. Microscopically, the lesion consisted of tightly packed homogenous glandular structures which are supported by a single layer of myoepithelial cells with sparse intervening stroma.

**Conclusions:**

We describe a very rare case of giant tubular adenoma arising within an accessory breast in the anterior chest wall in a late pregnancy woman. The high concentrations of estrogen, progesterone and prolactin might account for the significant tumor enlargement during pregnancy. To our knowledge, this is the first case of giant tubular adenoma occurred within the accessory breast in the anterior chest wall.

**Virtual slides:**

The virtual slide(s) for this article can be found here: http://www.diagnosticpathology.diagnomx.eu/vs/6210811191552106.

## Background

Tubular adenoma of the breast is a rare benign epithelial tumor and only a few literatures have been reported, most of them are identified in young women of reproductive age [1], but no cases of tubular adenoma occurred in the accessory breast have been reported in the English literature. Tubular adenoma, also known as pure adenoma, accounts for 0.13 - 1.7% of benign breast lesions [2]. It was first recognized as a distinctive entity in 1968 by Persaud who referred to it as “fibroadenoma with predominantly glandular component” [3]. Clinically, it is difficult to differentiate tubular adenoma from other benign lesions (fibroadenoma) and from malignant breast cancer (tubular carcinoma) radiographically [4], and surgical excision is often necessary in order to reach a precise diagnosis and a definitive treatment [5]. We herein describe a very rare case of a rapidly enlarging mass arising within an accessory mammary gland in the anterior chest wall in a 26-year-old woman at 37 weeks of pregnancy with successful pregnancy outcome.

## Case presentation

A 26-year-old woman (gravid 4, para 1) at 37 weeks of pregnancy with rapid enlargement in left anterior chest wall during pregnancy was admitted to our breast surgery unit. She first noticed a peanut size palpable accessory breast lesion at 10 weeks of pregnancy and during pregnancy the mass gradually increased along with pain. A similar symptom occurred in her last pregnancy 5 years ago, but the mass in left anterior chest wall was limited in the peanut size during the whole gestation period. A delayed surgical intervention was suggested. But the lump gradually regressed and disappeared after delivery.

**Figure 1:**
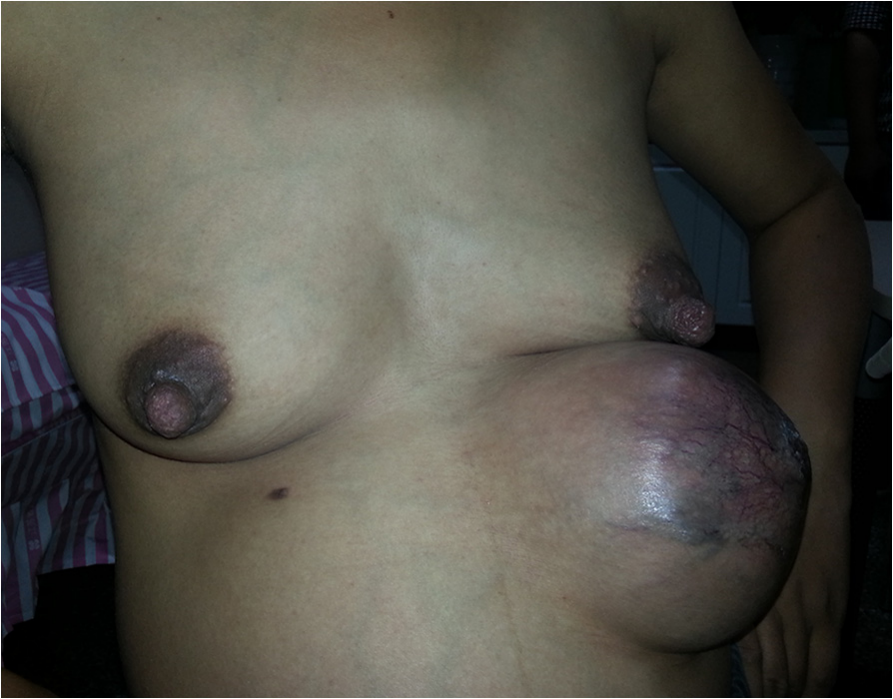
An elastic, mobile well-circumscribed mass in the left chest wall and left abdominal wall was seen. Due to progressive enlargement of the lump, diabrosis with bleeding occurred on the third day after admission.

On breast examination, two breasts looked symmetric with a couple accessory breasts below. The left accessory breast was obviously bigger than the right one that only had a light areola around a small nipple. An elastic, mobile well-circumscribed mass measuring approximately 15 cm × 15 cm was palpated on physical examination of the left accessory breast. Moreover, it was edematous and congestive with an increase in local temperature (Figure 1). The breast ultrasound further demonstrated the mass was a relatively homogeneous solid with short stripe blood flow signal. A single live fetus of 37 weeks gestation was observed by abdominal ultrasound scan. So mammography and dynamic contrast-enhanced Magnetic Resonance Imaging(MRI) were not performed. The patient also refused the core needle biopsy.

**Figure 2:**
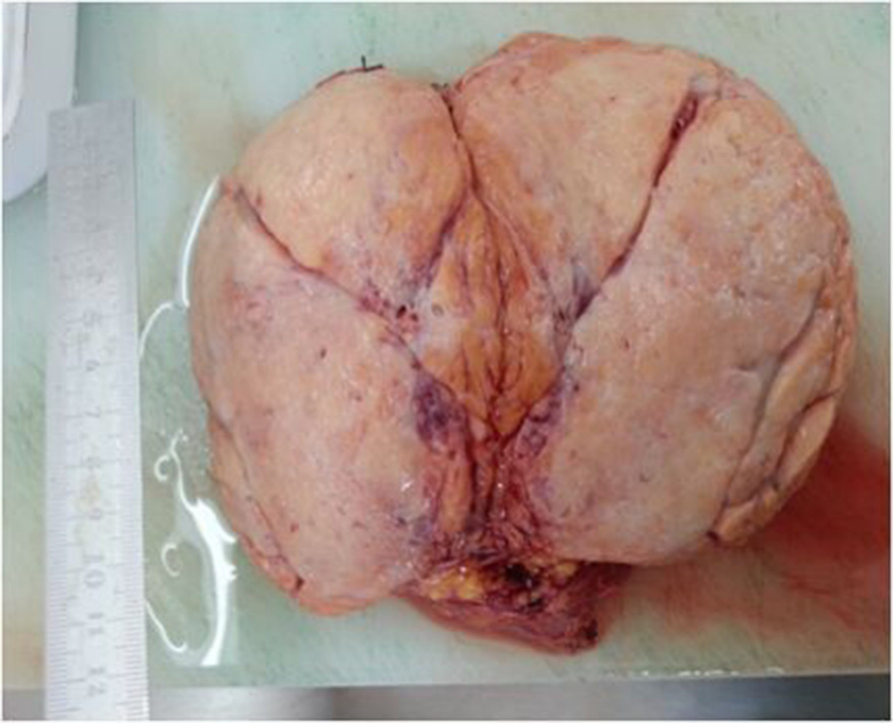
The resected specimen appeared as a solid white elastic mass with a smooth surface measuring approximately 15 cm × 15 cm × 12 cm. The cut surface was red-grayish.

**Figure 3:**
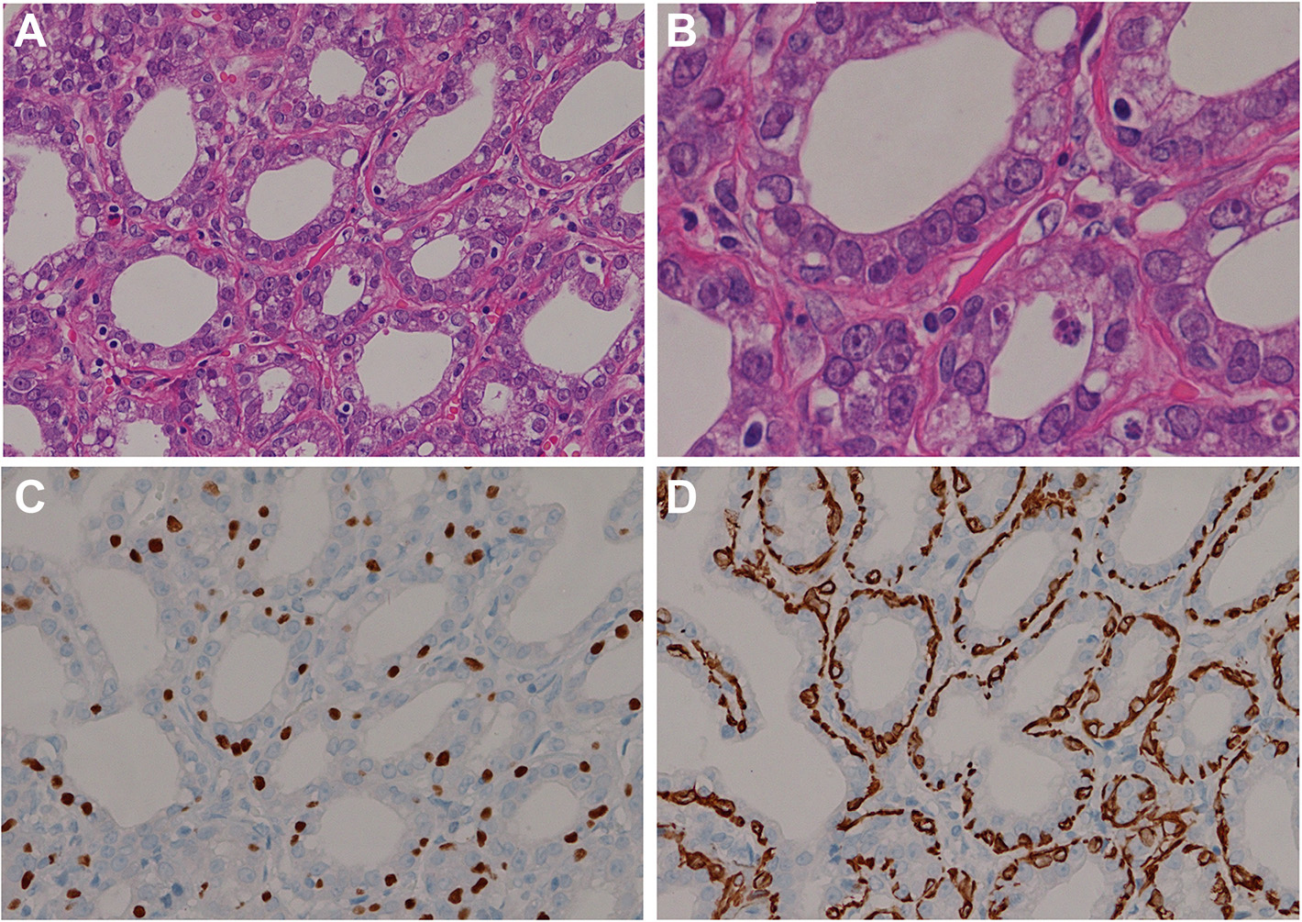
Microscopically, the lesion consisted of tightly packed tubular or acinar structures that were very regular in size and shape with sparse intervening stroma and the glandular structures were supported by a single layer of myoepithelial cells. (**A**: H&E × 200, **B**: H&E × 200, **C**: p63 × 200, **D**: calponin × 200).

Due to progressive enlargement of the lump, diabrosis with bleeding occurred on the third day after admission. Since the fetus had been mature in the 37th week of gestation, we decided to perform the excision of the mass and accessory breasts after caesarean section. After a 2850 g male neonate was delivered, the right accessory breast and the mass with left accessory breast were removed. The pathological result came out with a giant tubular adenoma of 15 cm × 15 cm × 12 cm, the resected specimen appeared as a solid white elastic mass with a smooth surface. The cut surface was red-grayish. No hemorrhage or necrosis was present (Figure 2). Microscopically, the lesion consisted of tightly packed homogenous glandular structures which were supported by a single layer of myoepithelial cells with sparse intervening stroma (Figure 3). Milk overflowed from the left chest wall incision three days after operation and cured two days after the administration of bromocriptine. The postoperative course was uneventful and she discharged in good condition in two weeks.

## Discussion

Numerous cases of masses arising in accessory breast have been reported, including both benign and malignant lesions. The most common lump identified in accessory breast tissue is fibroadenoma, and there are scattered case reports of other tumors including phyllodes tumor and mammary carcinoma [6]. Most of them take place in the axilla, in accordance with the incidence of accessory breast tissue. Tubular breast adenoma is a rare benign epithelial lesion which is most commonly found in young women of reproductive age [1]. Some scholars have reported a few cases and described them as elastic-hard, smooth-surfaced, freely movable and well-circumscribed masses without associated skin or nipple alterations [1,3,7-11]. The clinical features and the ultrasound examination are similar to those of fibroadenoma and nonspecific. Therefore, pathological examination is usually necessary in order to reach a precise diagnosis and a definitive treatment. Histologically, tubular adenoma is characterized by the presence of tightly packed tubular or acinar structures that are very regular in size and shape with sparse intervening stroma on the contrary to firoadenoma which contains abundant stroma [12,13]. However, sometimes tubular adenoma and fibroadenoma could coexist in one lesion, suggesting that the pathogenesis is closely related [11]. Apart from fibroadenoma, phyllodes tumor which also clinically present as painless freely movable well-defined breast masses should be considered as another differential diagnosis due to the possibility of malignancy [14]. In rare cases, the elastic-hard and rough-surfaced mass could also be the presence of malignancy, especially a mass with rapid enlargement in elderly post-menopausal women [15]. Soo MS compared the imaging features of 17 patients and found that tubular adenoma may resemble a malignancy in patients who were 38 years old or older [4]. Awareness of this may be helpful in preventing aggressive treatment.

Although there is no evidence for the correlation between the occurrence of tubular adenoma and pregnancy, tubular breast adenoma increases in size during pregnancy and could show lactational histologic changes [16]. The high concentrations of estrogen, progesterone and prolactin which promote the growth of ducts and the formation of tubuloalveolar structures might be a reason for the significant tumor enlargement in this period.

During gestation and lactation, the breast undergoes extensive changes and could create diagnostic challenges. Due to high sensitivity and specificity, ultrasound imaging was concerned to be the first choice [17]. Besides, MRI is considered to be adopted, although some literatures reports that Gadolinium-based contrast medium can pass through the placenta [18]. In the diagnostic process, fine-aspiration biopsy (FNAB) and core biopsy remains an increasing important diagnostic tool to evaluate the mass. Except for injuring the pregnancy and the fetus, FNAB and core biopsy provides a certain amount of samples for pathological examination. However, it is noticeable that the false-positive rate and the false-negative rate of the pathological findings is higher due to the breast/accessory breast changes in pregnancy [19], furthermore, core biopsy could induce fistula formation [20]. Breast or accessory breast surgery can be safely performed in whole gestation stage. However, because the risk of spontaneous abortion increases in first trimester [21], we suggest operation should be performed after the first trimester of gestation.

In this case, leakage of milk from the left chest wall incision happened three days after the stimulation of breastfeeding because of the injury to the milk duct during the operation. To avoid this, it is important to be more careful when deal with the edge of tumor nearby normal breast tissues. Some more powerful surgical instruments which could seal the duct also may be helpful. Cessation of breast feeding is necessary along with pharmacological suppression of lactation to prevent continuous milk overflowing from the incision.

## Conclusions

We describe a very rare case of giant tubular adenoma arising within an accessory breast in the anterior chest wall in a late pregnancy woman. The high concentrations of estrogen, progesterone and prolactin might account for the significant tumor enlargement during pregnancy. To our knowledge, this is the first case of giant tubular adenoma occurred within the accessory breast in the anterior chest wall. Doctors should be aware that diseases of the breast may also arise in accessory mammary tissue no matter how rare the disease is.

## Consent

Written informed consent was obtained from the patient for publication of this case report and any accompanying images. A copy of the written consent is available for review by the Editor-in-Chief of this journal.

## Abbreviations

MRI: Magnetic Resonance Imaging
FNAB: Fine-aspiration biopsy
H&E: Hematoxylin-eosin staining
